# Laparoscopic Hiatal Hernia Repair in Patients with an Intrathoracic Pancreas: Case Series and a Review of Literature

**DOI:** 10.7759/cureus.7125

**Published:** 2020-02-28

**Authors:** Robert C Tolboom, Paul J Wijsman, Ivo Broeders, Werner A Draaisma

**Affiliations:** 1 Surgery, Meander Medical Center, Amersfoort, NLD; 2 Anesthesiology, Radboud University Medical Center (Radboudumc), Nijmegen, NLD; 3 Robotics and Mechatronics, University of Twente, Enschede, NLD; 4 Surgery, Jeroen Bosch Hospital, Den Bosch, NLD

**Keywords:** thoracic, pancreas, hiatal hernia, type iv, pancreatic herniation

## Abstract

Transhiatal herniation of the pancreas is rare with only 17 cases reported in 25 years. Presentation of pancreatic herniation is diverse. In the majority of cases, the pancreatic herniation is found incidentally on CT-scans made for evaluating complaints related to a large or giant hiatal hernia. We present a literature review and case series of three patients with symptomatic type IV hiatal hernia with incidental, asymptomatic pancreatic herniation. All cases were managed laparoscopically with robotic assistance.

## Introduction

Hiatal hernia is either congenital or acquired. Acquired hiatal hernia can be classified based upon the position of the gastro-esophageal junction and the extent of herniation. The most common (95%) hernia is a type I or sliding hernia. In these hernias only the gastro-esophageal (GE) junction is herniated into the chest. A type II hernia is characterized by herniation of the gastric fundus into the mediastinum adjacent to the esophagus with the GE-junction still in the correct position. In type III (mixed) hiatal hernia, at least 30% of the stomach is herniated along with the GE-junction [1,2]. Only a type IV giant hiatal hernia (0.3%) and congenital hernias (0.2%) are characterized by herniation of additional organs besides the stomach. The most frequently herniated organs are the colon, small intestine, omentum, or spleen [3,4].

While small hiatal hernia is common and often without symptoms, large and giant hiatal hernias are less frequently seen and may present themselves with a myriad of complaints ranging from gastro-esophageal reflux and regurgitation to symptoms of heart failure due to cardio-pulmonary compression [1]. Though intraperitoneal organs such as the transverse colon sometimes herniate along with the stomach, herniation of the pancreas is very rare.

We present a series of three patients with pancreatic herniation, and discuss all available literature in order to advise on (surgical) management of this rare condition.

## Case presentation

Patient A

**Figure 1:**
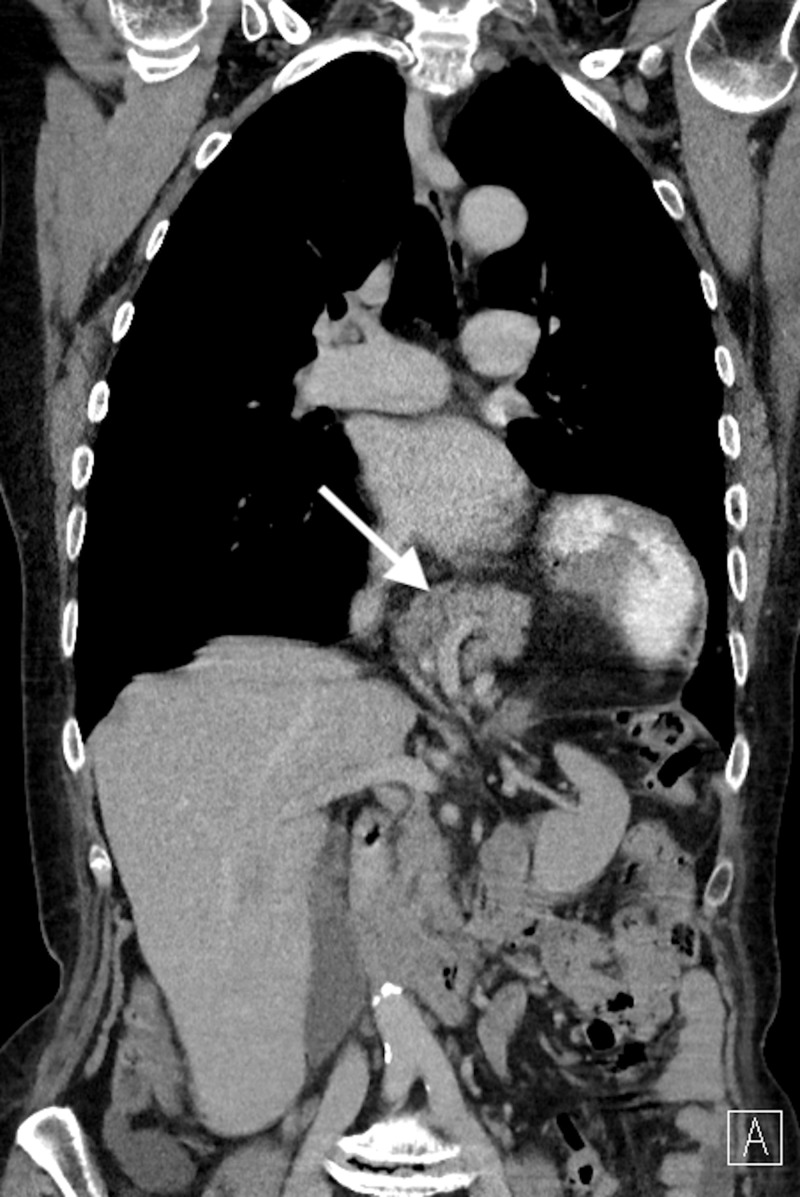
CT-scan of Patient A depicting herniation of the pancreas into the thoracic cavity.

A 74-year-old woman with a history of ischemic stroke, transabdominal uterus extirpation and left inguinal hernia repair was referred for outpatient evaluation after several years of dyspnea, dyspepsia, vomiting and thoracic pain. Laboratory studies were within normal limits. Computed tomography (CT) examination showed herniation of the stomach and pancreas through the esophageal hiatus (Figure 1) without any pancreatic pathologic findings or signs of inflammation. Esophagogastroduodenoscopy excluded the presence of esophagitis.

With the diagnosis of a symptomatic type IV (giant) hiatal hernia, an elective robot-assisted laparoscopic surgery was planned. After intubation, the patient was placed in a reverse Trendelenburg and French position with both arms extended. Five ports were used and introduced in a smiley-face configuration. The da Vinci robot was docked from over the head. Several adhesions were taken down followed by introduction of an endopaddle to retract the left lateral segment of the liver superiorly. Using a hook cautery, the hiatus was successfully dissected. The stomach and pancreas could easily be repositioned intra-abdominally after which the crus was closed with two posterior sutures, followed by one anteriorly. All stitches were reinforced with 1 x 1 cm polypropylene pledgets. To conclude the procedure, an anterior fundoplication was made. The total duration of the uncomplicated procedure was 90 minutes.

The postoperative course was uneventful and the patient was discharged on the fifth postoperative day. Two years after surgery, the patient developed a spontaneous recurrent hiatal hernia with complaints of dyspnea which was corrected, and a pyloromyotomy was performed because of gastric emptying disorders seen on fasted computed tomography scan. Three years after surgery, she is in good condition, with limited dyspeptic symptoms.

Patient B

**Figure 2:**
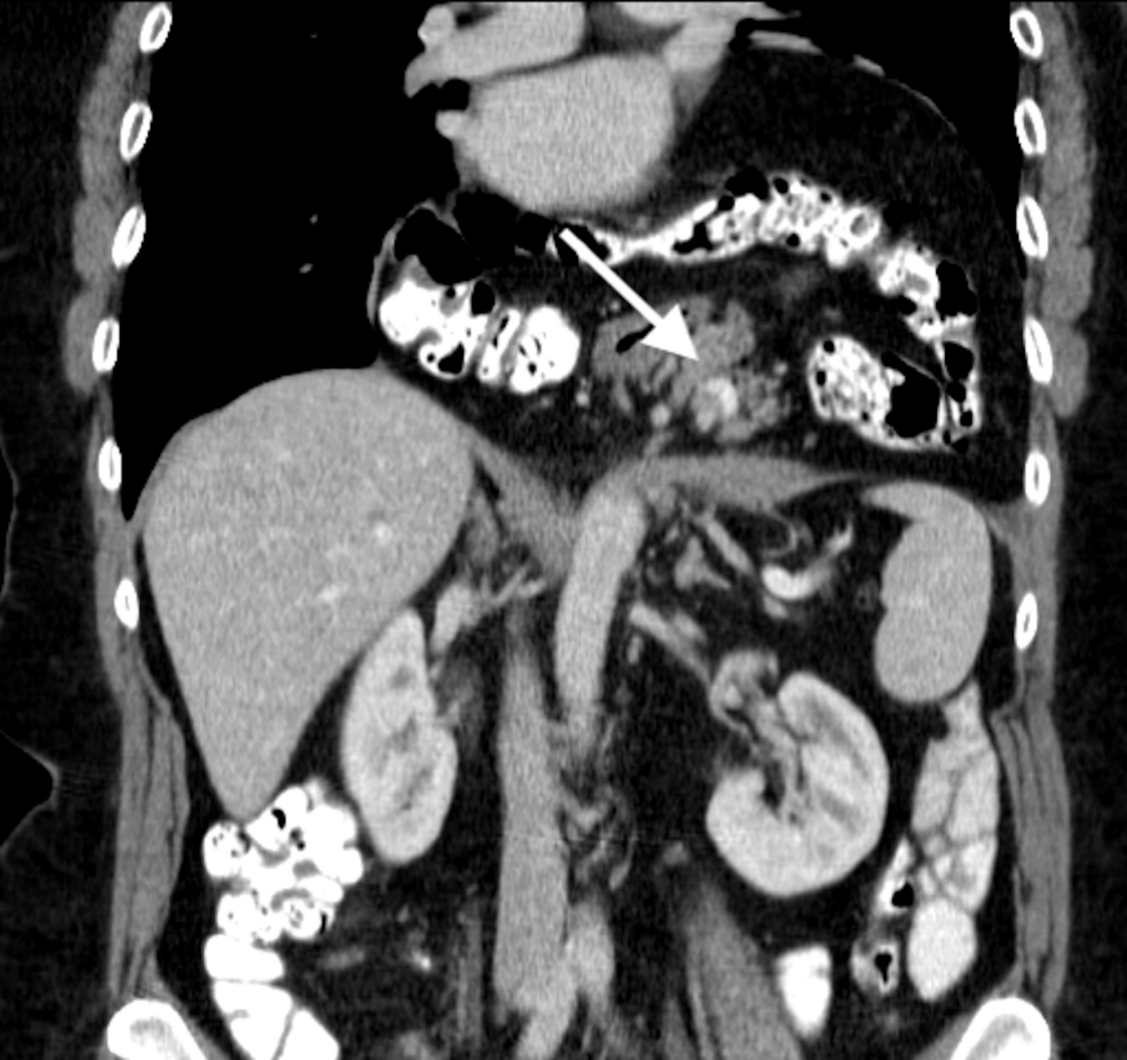
CT-scan of Patient B depicting herniation of the pancreas into the thoracic cavity.

A 66-year-old woman with a history of a laparoscopic cholecystectomy was referred for outpatient evaluation due to progressive complaints of pressure on the chest and shortness of breath after exertion without chest pain. These complaints gradually worsened in a period of two years to the point where the patient was no longer able to walk, ride her bike or sing. She also suffered from dyspepsia. A CT-scan revealed a giant hiatal hernia with complete intrathoracic stomach, transverse colon and pancreas (Figure 2).

An elective robot-assisted laparoscopic hiatal hernia repair was planned. After crural dissection, the stomach, transverse colon and pancreas were reduced. Due to the hernia's size, lateral incisions were necessary before a PROCEED® mesh-reinforced crural closure could be attempted. With the lateral incision, the thoracic cavity was opened. This defect was later bridged using a moon-shaped PROCEED® surgical mesh using V-Loc sutures. Due to expected pleural effusion, a chest drain was placed. After crural closure, an anterior fundoplication was made. Duration of surgery was 135 minutes.

The post-operative course was uneventful. The chest drain was removed the first post-operative day, and the patient discharged on the fourth. At long-term follow-up, all complaints were resolved.

Patient C

**Figure 3:**
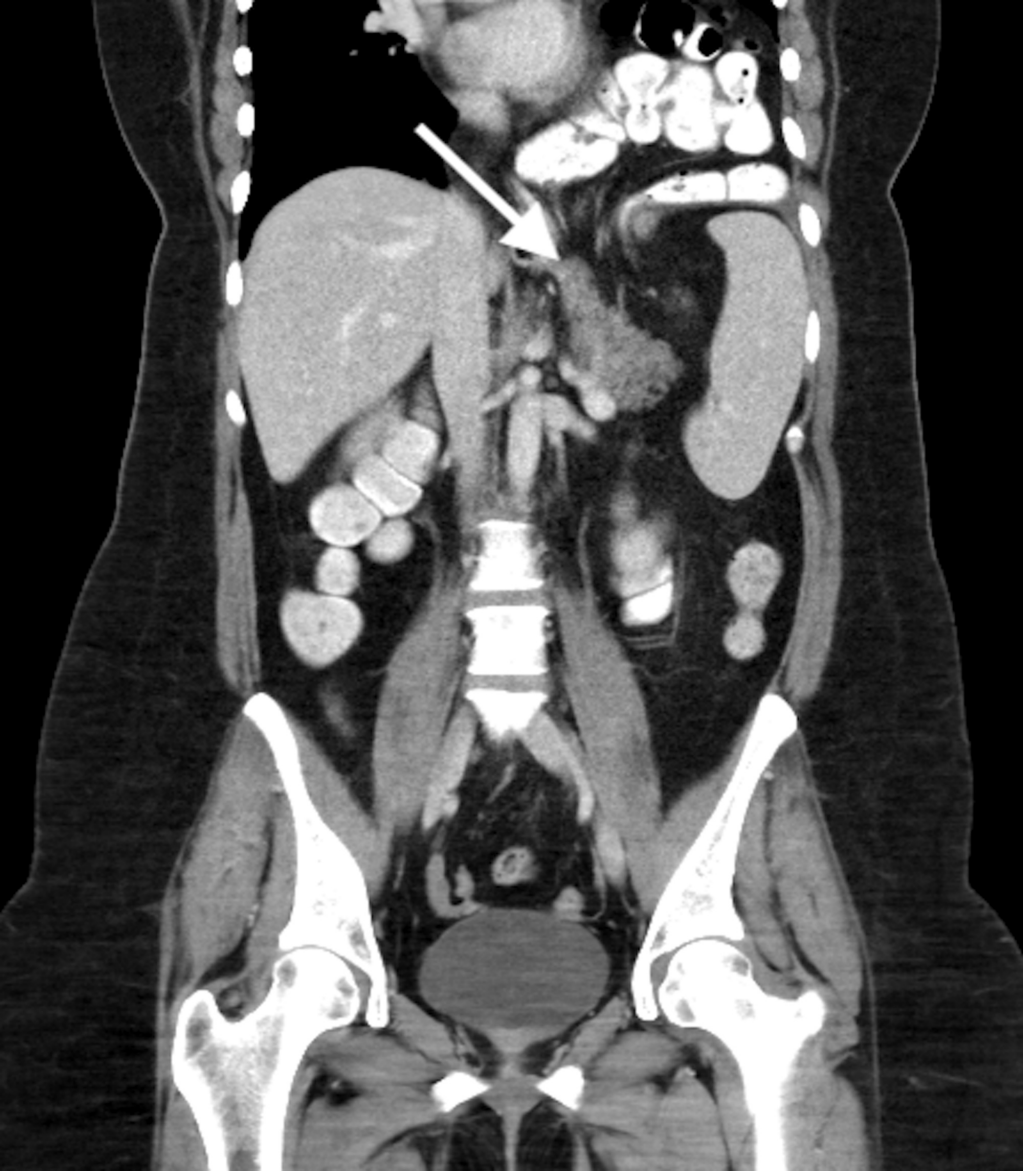
CT-scan of Patient C depicting herniation of the pancreas into the thoracic cavity.

A 42-year-old woman with a history of a transvaginal hysterectomy, laparoscopic cholecystectomy, epilepsy and chronic depression was referred to our outpatient clinic due to complaints of nausea, recurrent vomiting, abdominal and retrosternal pain and dyspepsia. Laboratory studies were within normal limits. A CT-scan showed a large hiatal hernia (>7 cm) with intrathoracic stomach, colon and pancreas (Figure 3).

In an elective robot-assisted laparoscopic hiatal hernia repair lasting 110 minutes, the stomach, pancreas and colon were reduced. The crus was closed with two stitches posteriorly and three stitches anteriorly, followed by anterior partial fundoplication.

After an uneventful postoperative course, the patient was discharged on the seventh post-operative day. At long-term follow-up she had moderate dyspeptic symptoms. No signs of recurrent hiatal hernia were seen on follow-up CT scans.

## Discussion

The pancreas is formed in the dorsal mesoduodenum from the endodermal germ layer and will be positioned in the dorsal mesogastrium at the end of the fifth gestational week. Meanwhile, the stomach rotates and grows disproportionately. By rotating about the longitudinal axis, the dorsal mesogastrium is pulled to the left, creating a space behind the stomach called omental bursa (or lesser peritoneal sac). This rotation also pulls the ventral mesogastrium to the right. With continued rotation of the stomach, the dorsal mesogastrium lengthens, and partly fuses with the peritoneum of the posterior abdominal wall. The tail of the pancreas lies against this region. After degeneration of the posterior leaf of the dorsal mesogastrium and peritoneum of the posterior body wall along the line of fusion, the pancreatic tail is covered by peritoneum on the anterior surface only and therefore lies in a retroperitoneal position [5].

When the colon and small intestine are herniated, stretching of transverse mesocolon may allow mobilization of the pancreas after lengthening of the posterior adhering fascia [2,6]. In a recent human cadaver study, researchers found retropancreatic fascia lining the posterior aspect of the body of the pancreas but were unable to locate the anterior renal fascia, possibly due to degeneration of the adrenal gland with ageing [7]. It is likely that in the elderly the pancreas is more mobile due to connective tissue degeneration, and therefore more prone to migrating through a large hiatal hernia [7].

In the last 25 years, 17 cases of intrathoracic herniation of (parts of) the pancreas have been reported (Table 1) [2-4,6,8-20]. In the majority of cases, the pancreatic herniation itself was asymptomatic and found incidentally [2,4,8-12,16-20] on CT-scans made for evaluating complaints befitting a large or giant hiatal hernia such as abdominal pain [4,10,11,16-19], vomiting [17,18], dysphagia [4] or dyspnea [2,16,20]. Five cases, however, were diagnosed with pancreatitis secondary to intrathoracic pancreatic herniation [3,6,13-15], one of which was associated with pancreatic torsion [6].

**Table 1:** Literature review: transhiatal pancreatic herniation.

Author	Year	Age (yr)	Sex	Presentation	Symptoms					Diagnosis						Treatment
					Abdominal pain	Vomiting	Dyspnoea	Palpitations	Dysphagia	Hernia type	Herniated part(s)	Other herniated organs	Pancreatitis	Volvulus	Incarceration	
Oliver et al. [15]	1990	5	M	Emergency department	+	+	-	-	-	Congenital (Bochdalek)	Tail	Spleen, colon, left kidney and adrenal gland	+	+	-	Laparotomy
Chevallier et al. [6]	2001	70	M	Emergency department	+	-	-	-	-	Acquired (large/giant)	Body & Tail (transient)	Stomach	+	+	-	Conservative initially; elective surgery
Saxena et al. [16]	2006	78	F	Emergency department	+	-	+	-	-	Acquired (large/giant)	Body & Tail	(not reported)	-	-	-	Thoracotomy
Tagaya et al. [19]	2007	75	F	Emergency department	+	-	-	-	-	Acquired (large/giant)	Body & Tail	Stomach, colon and jejunum	-	-	+	Laparoscopy
Soylu et al. [18]	2010	26	M	Outpatient clinic	+	+	-	-	-	Congenital (Bochdalek)	Tail	Spleen, stomach, small intestine and transverse colon	-	-	+	Laparotomy
Ahmed et al. [9]	2010	57	M	Routine follow-up	-	-	-	-	-	Acquired after transhiatal esophagectomy	Body & Tail	(not reported)	-	-	-	Conservative
Coughlin et al. [4]	2011	61	F	Outpatient clinic	+	-	-	-	+	Acquired (large/giant)	Mid-body	Stomach	-	-	-	Elective laparotomy
Torres et al. [20]	2013	82	F	Emergency department	-	-	+	-	-	Acquired (large/giant)	Body & Tail	Stomach	-	-	-	Conservative
Valente et al. [2]	2013	68	M	Emergency department	-	-	+	-	-	(not reported)	Mid-body	None	-	-	-	(not reported)
Shah et al. [17]	2013	53	F	Emergency department	+	+	-	-	-	Unknown	Whole	Stomach, transverse colon and spleen	-	-	-	Laparoscopy
Kumar et al. [13]	2013	89	M	Emergency department	+	+	-	-	-	(not reported)	Mid-body	None	+	-	-	Conservative
Hadjittofi et al. [11]	2013	17	M	Emergency department	+	-	-	-	-	Congenital (Bochdalek)	Tail	Stomach and spleen	-	+	-	Laparoscopy
Jäger et al. [12]	2013	72	F	Emergency department	-	-	-	-	-	Acquired (large/giant)	Neck & Body	Duodenum	-	-	-	Conservative initially; elective laparotomy
Agrawal et al. [8]	2014	32	M	Routine follow-up	-	-	-	-	-	Acquired after transthoracic esophagectomy	(not reported)	None	-	-	-	Conservative
Boyce et al. [3]	2014	61	F	Emergency department	+	+	+	-	-	Acquired (large/giant)	Neck & Body	Stomach, colon and small intestine	+	-	+	Conservative initially; elective laparotomy
Lu et al. [14]	2015	88	M	Emergency department	+	-	-	-	-	Acquired (large/giant)	Body & Tail (transient)	Stomach and duodenum	+	-	-	Conservative
Furtado et al. [10]	2015	59	M	Emergency department	+	-	-	-	-	Acquired (large/giant)	Neck & Body	Stomach and duodenum	-	-	-	Laparoscopy

While pancreatitis is known to occur as a result of pancreatic herniation, large and giant hiatal hernia without pancreatic involvement may also present with acute or recurrent pancreatitis. The underlying mechanism is believed to be that of intermittent ischemia due to traction on the vascular pedicle [3,14,15], repetitive trauma associated with pancreatic movement across the hernia [3] or torsion with occlusion of the main pancreatic duct [6].

Pancreatic herniation may occur in patients with many different kinds of hiatal hernia. They have been reported in congenital [11,15,18] and acquired hernias [3,4,6,10,12,14,16,19,20], but also as rare long-term complication after both transhiatal and transthoracic esophagectomy [8,9]. Even isolated (possibly congenital) herniation of just the pancreas [2] and transient pancreatic herniation have been reported [6,14].

Treatment decisions should be made based upon patient presentation, type of hiatal hernia and herniated organs. All symptomatic hiatal hernias may be considered for surgery while completely asymptomatic acquired hiatal hernia with herniation of one or more organs including the pancreas may be managed conservatively with watchful waiting. In case of presentation with acute or recurrent pancreatitis, delayed surgery should be attempted due to an increased morbidity and mortality associated with the systemic inflammatory response.

The approaches available for reduction of the herniated organs and hiatal hernia repair include transthoracic, transabdominal, and laparoscopic. In our case series we demonstrate that in the absence of pancreatitis, large and giant hiatal hernia with pancreatic involvement may be safely managed laparoscopically (possibly with robotic assistance). Two patients in this small series had persistent dyspeptic symptoms. This might be related to the extensive dissection and stretching of the retracted esophagus, and damage to vagal nerve branches despite the meticulous dissection to avoid nerve disruption or thermal damage.

## Conclusions

This case series adds to the existing evidence that laparoscopic surgery for giant hiatal hernia with herniation of the pancreas is feasible and safe, allowing patients to quickly resume their daily activities.
